# Inhibition of Dephosphorylation of Dolichyl Diphosphate Alters the Synthesis of Dolichol and Hinders Protein *N*-Glycosylation and Morphological Transitions in *Candida albicans*

**DOI:** 10.3390/ijms20205067

**Published:** 2019-10-12

**Authors:** Anna Janik, Monika Niewiadomska, Urszula Perlińska-Lenart, Jacek Lenart, Damian Kołakowski, Karolina Skorupińska-Tudek, Ewa Swiezewska, Joanna S. Kruszewska, Grażyna Palamarczyk

**Affiliations:** 1 Institute of Biochemistry and Biophysics, Polish Academy of Sciences, Pawinskiego 5a, 02-106 Warsaw, Poland; annaj@ibb.waw.pl (A.J.); monikaniew@ibb.waw.pl (M.N.); ulalenart@o2.pl (U.P.-L.); damian.kolakowski@ibb.waw.pl (D.K.); korolina@ibb.waw.pl (K.S.-T.); ewas@ibb.waw.pl (E.S.); gp@ibb.waw.pl (G.P.); 2 Mossakowski Medical Research Centre, Polish Academy of Sciences, Pawinskiego 5, 02-106 Warsaw, Poland; lenart@ibb.waw.pl

**Keywords:** *C. albicans*, cell wall integrity, Cwh8p, dolichol, morphogenesis, Rer2p

## Abstract

The essential role of dolichyl phosphate (DolP) as a carbohydrate carrier during protein *N*-glycosylation is well established. The cellular pool of DolP is derived from de novo synthesis in the dolichol branch of the mevalonate pathway and from recycling of DolPP after each cycle of *N*-glycosylation, when the oligosaccharide is transferred from the lipid carrier to the protein and DolPP is released and then dephosphorylated. In *Saccharomyces cerevisiae*, the dephosphorylation of DolPP is known to be catalyzed by the Cwh8p protein. To establish the role of the Cwh8p orthologue in another distantly related yeast species, *Candida albicans*, we studied its mutant devoid of the *CaCWH8* gene. A double *Cacwh8∆*/*Cacwh8∆* strain was constructed by the *URA*-blaster method. As in *S. cerevisiae*, the mutant was impaired in DolPP recycling. This defect, however, was accompanied by an elevation of *cis*-prenyltransferase activity and higher de novo production of dolichols. Despite these compensatory changes, protein glycosylation, cell wall integrity, filamentous growth, and biofilm formation were impaired in the mutant. These results suggest that the defects are not due to the lack of DolP for the protein *N*-glycosylation but rather that the activity of oligosacharyltransferase could be inhibited by the excess DolPP accumulating in the mutant.

## 1. Introduction

Dolichol synthesis is a multistep process whose initial stagesfrom the acetyl-CoA precursor up to farnesyldiphosphate (FPP)are a common part of the mevalonate pathway leading to the synthesis of all isoprenoids. The dolichyl branch commences with the activity of two *cis*-prenyltransferases (*cis*-PTase-Rer2p/Nus1p and Srt1p/Nus1p) sequentially elongating FPP with five-carbon moieties. The reaction involves (1′–4) head-to-tail condensation of isopentenyl pyrophosphate (IPP, C_5_) with FPP (C_15_), followed by further elongation with IPP. Finally, the resultant dehydro-dolichyl diphosphate with a defined, species-specific chain length (in *C. albicans* C_70_–C_90_) is dephosphorylated and reduced by alfa-saturase (provisionally identified as Dfg10p [1]) to form dolichol. To enter the glycosylation pathway, dolichol undergoes phosphorylation by the CTP-dependent dolichyl kinase Sec59p [2,3].

The importance of DolP in eukaryotes is well established. Crucially, its availability modulates the level of protein glycosylation. It has been proposed that in addition to the de novo dolichol synthesis, the DolP pool is enriched by the DolP released from the DolPP-oligosaccharide precursor after each cycle of glycosylation [4]. Indeed, when the precursor oligosaccharide is transferred from the lipid carrier to an appropriate AsnXSer/Thr sequence in the nascent polypeptide, DolPP is released in the ER lumen and partially dephosphorylated to give DolP. A DolPP phosphatase with a lumen-oriented active site, responsible for converting DolPP to DolP, has been identified in yeast (Cwh8p) and in mammalian cells (DolPP1p) [4,5,6]. Moreover, since in a *cwh8∆* yeast mutant the capacity to form DolPP-oligosaccharide was reduced, it was assumed that the de novo synthesis of DolP is not sufficient to support the cell requirement for DolP and, at least in some systems, the recycling of DolP after glycosylation is needed to ensure its correct level. 

Notably, numerous studies on the physiological role of free dolichol have suggested that also the alcohol backbone of DolP could modulate the key membrane properties, fluidity and permeability, and cause extensive membrane trafficking between the Golgi system, the plasma membrane, and the lysosomes [7,8,9]. To address this suggestion and to study the effect of perturbed DolP recycling on cell functioning, we used another model system, the yeast *Candida albicans*. 

The *C. albicans* genome contains open reading frames *orf 19.4028* and *orf 19.5236* encoding proteins with significant similarity to the *S. cerevisiae* Rer2p and Srt1p proteins [10]. In addition, a homologue of the *S. cerevisiae NUS1* gene can be found in the *C. albicans* data base. All these proteins are *cis*-prenyltransferases in *S. cerevisiae* [11]. Moreover, according to the *C. albicans* genome data base, *orf 19.3682* encodes a Cwh8p protein with a high similarity to the *S. cerevisiae* DolPP phosphatase. 

We have previously demonstrated that the perturbation of dolichol synthesis following the suppression of the *RER2* gene alters the integrity of the cell wall and prevents hyphae formation in *C. albicans*. Moreover, a similar effect was observed upon mutation of the *ALG13* gene encoding *N*-acetylglucosaminyl (GlcNAc) transferase, a subunit of the protein complex synthesizing DolPPGlcNAc_2_ which initiates protein *N*-glycosylation [12]. 

In the present work we extended those studies by determining the effect of disrupted DolPP recycling due to *CWH8* deletion on the functioning of the *C. albicans* cells. We were particularly interested in protein glycosylation, cell wall properties, and de novo synthesis of dolichol. 

## 2. Results

### 2.1. C. albicans Orthologue of the S. cerevisiae CWH8 Gene

**Figure 1:**
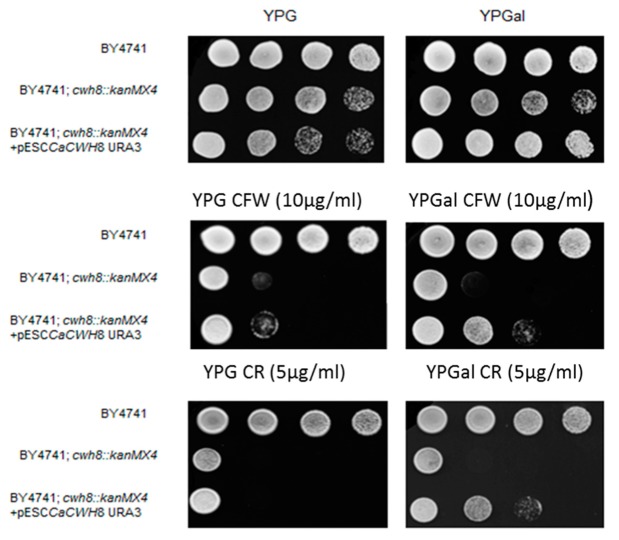
*C. albicans CWH8* gene partially restores resistance of *S. cerevisiae* BY4741 *cwh8: kanMX4* to Calcofluor White and Congo Red.

The *C. albicans* genome contains an open reading frame *orf 19.3682* whose putative protein product shows 42.6% identity with Cwh8p of *S. cerevisiae*. As postulated earlier, the Cwh8p protein is involved in DolPP/DolP recycling, needed for the reutilization of the DolP in subsequent rounds of DolPP-oligosaccharide biosynthesis and protein *N*-glycosylation [4]. To check if the *C. albicans* gene encodes a functional homologue of Cwh8p, we used it to complement the cell wall defect of an *S. cerevisiae cwh8Δ* mutant caused by defective protein *N*-glycosylation. The coding region of *orf 19.3682* was ligated into the pESC yeast vector under control of the *GAL1* promoter and introduced into *S. cerevisiae cwh8Δ*. Expression of the construct significantly decreased the sensitivity of the defective strain to Calcofluor White and Congo Red (Figure 1). 

Serial 1:10 dilutions of yeast suspension were spotted on YPG and YPGal plates and cultivated at 30 °C for 72 h. Induction of *CaCWH8* expression from *GAL1* promoter on medium containing 2% galactose rendered *S. cerevisiae cwh8Δ* cells more resistant to Calcofluor White (CW) and Congo Red (CR).

This complementation showed that *orf 19.3682* of *C. albicans* does indeed encode a homologue of the *S. cerevisiae* Cwh8p capable of recycling DolPP. We therefore propose to name *orf 19.3682 CaCWH8*. 

### 2.2. Homozygous Disruption of the C. albicans CWH8 Gene Increases Dolichol Level

**Figure 2:**
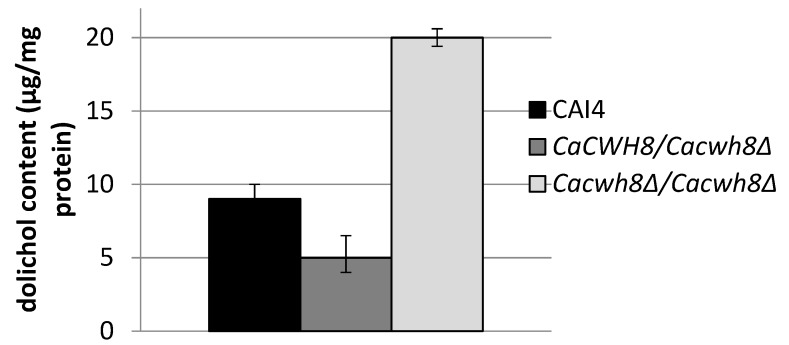
Dolichol content in the wild type *C. albicans* strain CAI4 and *CaCWH8* mutants. Data are mean ± standard deviation from three independent experiments.

To study the participation of CaCwh8p in dolichol metabolism in *C. albicans* we constructed a homozygous deletion strain *Cacwh8∆*/*Cacwh8∆*. Unexpectedly, quantitative analysis of dolichol showed that the mutant contained roughly double the amount of the dolichols found in the parental wild type strain. In contrast, in the *CaCWH8*/*Cacwh8∆* hemizygote the dolichol content was diminished by 45% (Figure 2).

**Figure 3:**
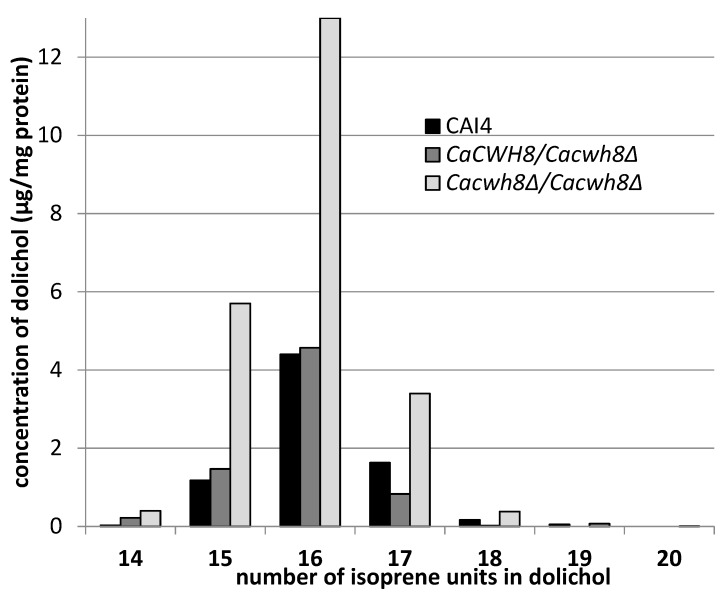
Content of dolichols of defined chain length in the wild type *C. albicans* strain CAI4 and the *CaCWH8* mutants.

A detailed analysis of dolichols from the wild-type *C. albicans* CAI4 strain and *Cacwh8∆*/*Cacwh8∆* mutant showed identical molecular species, similar to the dolichols found in *S. cerevisiae* [13]. The predominant dolichol contained 16 isoprene units, although shorter (14 and 15 isoprene units) as well as longer (up to 20 isoprene units) species were also found (Figure 3). 

In agreement with the general quantitative data above, this detailed analysis also indicated that the removal of both copies of the *CaCWH8* gene resulted in a doubling of the amount of dolichol compared to the wild-type control, elevating the level of the dominant dolichol species by almost three times.

**Figure 4:**
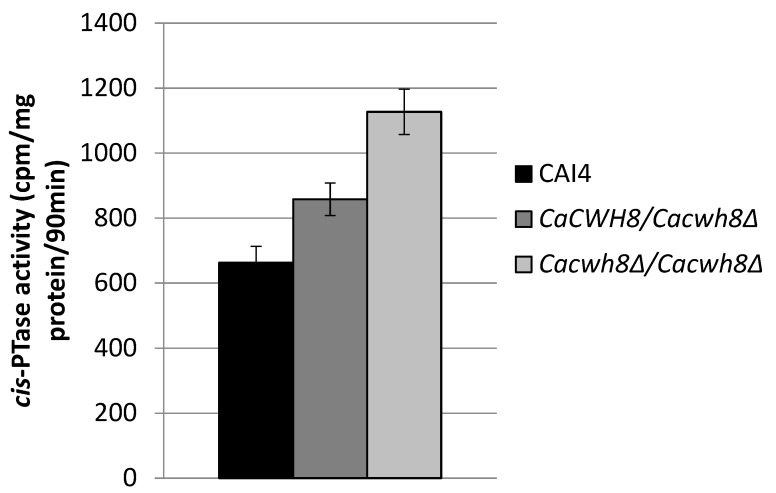
Activity of *cis*-PTase in the wild type *C. albicans* strain CAI4 and the *CaCWH8* mutants.

In order to check if the increased content of dolichol in the null mutant was due to an increased activity of *cis*-PTase, a key enzyme in dolichol synthesis, we determined its activity in the membrane fraction. It turned out to be higher by 70% in the *Cacwh8∆*/*Cacwh8∆* mutant compared to the control CAI4 strain (Figure 4). 

*cis*-PTase activity was measured in the presence of [^14^C] IPP and with exogenous FPP. The products of reaction were developed on RP18 HPTLC plates, detected by autoradiography and quantified by liquid scintillation counting. Data are mean ± standard deviation from three independent experiments. Unexpectedly, the activity of *cis*-PTase was also increased in the hemizygote (by 29%), although the level of dolichol was not elevated in this strain.

### 2.3. Cwh8p Protein Affects Transcription of Genes Encoding Proteins Presenting Cis-PTase Activity.

The synthesis of dolichols is catalyzed by two complexes of *cis*-PTases: Rer2p/Nus1p and Srt1p/Nus1p [13,14,15,16,17]. It has previously been shown that in *S. cerevisiae*, longer dolichols are synthesized by the Srt1p/Nus1p complex while shorter ones (16 and 17 isoprene units) appear during the logarithmic phase of growth when the Rer2p/Nus1p *cis*-PTase complex is active. 

While examining the dolichol pattern in the *C. albicans* mutants we found small amounts of longer dolichols containing 19 and 20 isoprene units in the *Cacwh8∆*/*Cacwh8∆* strain.

**Figure 5:**
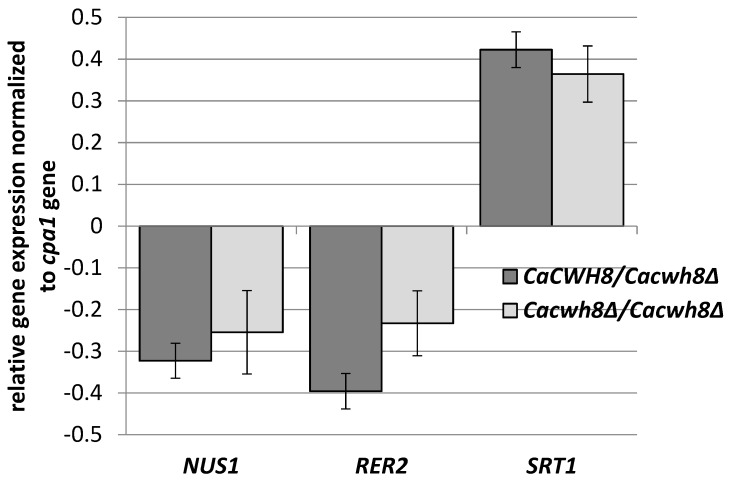
Relative transcript levels of *NUS1*, *RER2*, and *SRT1* genes in *C. albicans CaCWH8* mutants compared to the wild type strain.

Their increased content could indicate an enhanced activity of the Srt1p/Nus1p complex of *cis*-PTase in this strain; however, the amount of dolichol-16 in the *Cacwh8∆*/*Cacwh8∆* mutant was many times higher (Figure 3), suggesting that the activity of the Rer2p/Nus1p complex was dominant. To determine how the lack of the CaCwh8p DolPP phosphatase affects the individual *cis*-PTases, expression of *CaRER2*, *CaSRT1*, and *CaNUS1* genes in the wild type and the *Cacwh8* mutants was determined (Figure 5).

*C. albicans* strains were grown for 16 h in YPD medium, RNA was extracted, and cDNA synthesized. qPCR reactions were performed using LightCycler 1.6. The crossing point (Cp) value (cycle number in the log-linear region) was calculated using the LightCycler quantification software. Values were normalized against the transcript of the reference gene *CPA1* encoding carboxypeptidase 

Data are mean ± standard deviation from three independent experiments, each determined in triplicate.

Deletion of one or both copies of the *CaCWH8* gene decreased expression of the *CaRER2* and *CaNUS1* genes and increased the expression of *CaSRT1* (Figure 5). 

The elevated expression of *CaSRT1* in the *Cacwh8∆*/*Cacwh8∆* mutant could explain the appearance of longer dolichols in the cells of this strain. Furthermore, the total activity of *cis*-PTases in the *Cacwh8∆*/*Cacwh8∆* mutant was significantly elevated compared to the wild type control and this could explain the increased overall production of dolichols (Figure 2). The hemizygous strain showed a stronger decrease of expression of the *CaRER2* gene than the null mutant, and a 44% decrease of the dolichol level (Figure 2). Apparently, in the hemizygous strain, the dolichol synthesis could not be rescued by the higher level of the CaSrt1p *cis*-PTase. 

### 2.4. Lack of CaCwh8p Activity Affects N-Glycosylation 

It is known that proper level of protein glycosylation depends, among other factors, on the availability of DolP arising by phosphorylation of free dolichol by CTP-dependent dolichol kinase. The increased level of dolichol in the *Cacwh8∆*/*Cacwh8∆* mutant (Figure 2) could result in an excess of DolP for the glycosyl transferases initiating *N*- and *O*-glycosylation, *N*-acetylglucosaminyl (GlcNAc) transferase and DPM synthase, respectively. 

The process of *N*-glycosylation starts with the formation of DolPPGlcNAc_2_ catalyzed by the GlcNAc transferase complex (Alg7p, Alg13p, and Alg14p) [18].

In turn, during *O*-glycosylation DPM synthase initiates the synthesis of dolichyl phosphate mannose (DPM) from GDPMan and DolP and the mannosyl residue is then transferred to the hydroxyl group of a serine or threonine in the protein. The mannosyl residues from DPM are also used in DolPP-oligosaccharide formation during *N*-glycosylation. To investigate the effects of CaCwh8p depletion on these initial steps of glycosylation, activities of DPM synthase and GlcNAc transferase were analyzed without addition of exogenous DolP. In these conditions, the enzymes can only use the endogenous pool of DolP from the membrane fraction added as the source of the enzymes.

Incorporation of [^14^C] mannose from GDPMan into DPM was quantified by liquid scintillation counting. Data are mean ± standard deviation from five independent experiments. 

**Figure 6:**
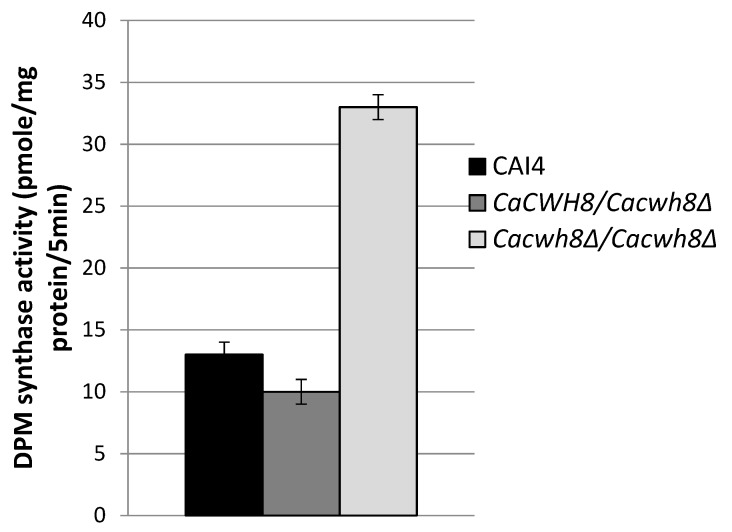
Activity of DPM synthase in membrane fractions from the wild type *C. albicans* strain CAI4 and the *CaCWH8* mutants.

As expected, the activity of DPM synthase was 2.5-fold higher in the *Cacwh8Δ*/*Cacwh8Δ* mutant (Figure 6) characterized with a much higher content of dolichol (Figure 2) compared to the control and the hemizygous strain. The lowest activity was observed for the hemizygote containing the lowest amount of dolichol in its membrane fraction. The high endogenous activity (without DolP added) of DPM synthase in the *CaCWH8* double mutant indicates that its membrane fraction contains more DolP than the other strain analyzed. 

Incorporation of N-acetyl [^14^C] glucosamine into DolPPGlcNAc and DolPPGcNAc_2_ was quantified by liquid scintillation counting.

**Figure 7:**
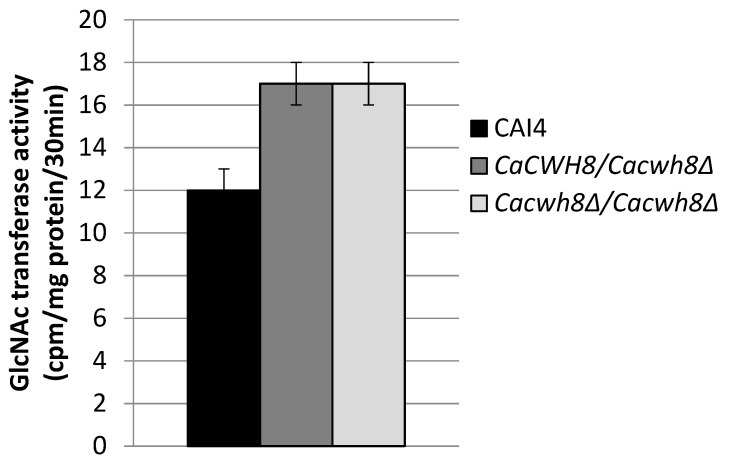
Activity of GlcNAc transferase in membrane fractions from the wild type *C. albicans* strain CAI4 and the *CaCWH8* mutants. Data are mean ± standard deviation from five independent experiments.

The endogenous (without DolP added) activity of GlcNAc transferase was 42% higher in both mutants compared to the control CAI4 strain (Figure 7). 

The elevated endogenous activities of both enzymes in the mutant strains suggested that protein glycosylation could occur without limitations. To verify this assumption, in vivo protein glycosylation was evaluated by determining the glycosylation status of the well-defined *C. albicans* glycoproteins Phr1p/Phr2p, which show52% and 56% identity, respectively, with the *S. cerevisiae* Gas1p, a GPI-anchored, *N*- and *O*-glycosylated β-1,3-glucosyltransferase involved in the cell wall remodeling [19,20]. Their expression is dependent on the pH of the growth environment: *PHR1* is expressed at a pH above 5.5, while for *PHR2*, the maximum of expression is observed below pH = 5.5 [19]. Since the final pH of the culture was higher than 5.5, we expect that the detected band corresponds to the Phr1p protein.

Aliquots of protein extracts were treated with Endo H to remove *N*-glycans or left untreated, and then analyzed by Western blotting. Rabbit anti-Gas1p primary antibody and HRP-conjugated anti-rabbit secondary immunoglobulin were used to detect Phr1p protein. Antibody–protein complex was visualized with chemiluminescent substrate. 

**Figure 8:**
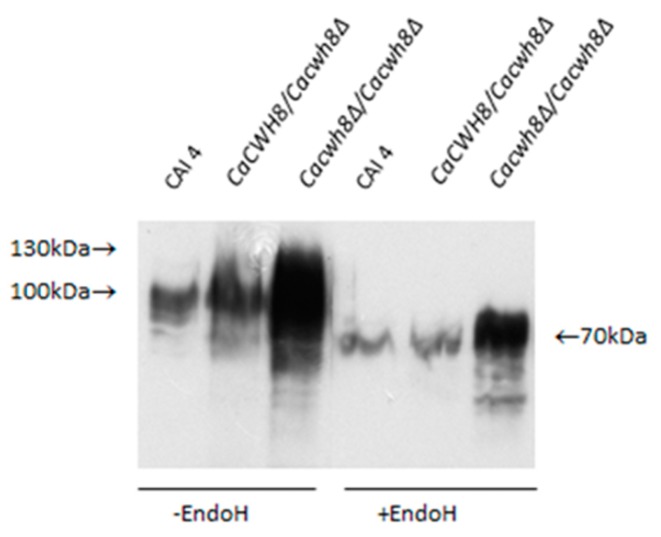
*N*-glycosylation of Phr1p protein in the wild type *C. albicans* strain CAI4 and the *CaCWH8* mutants. Proteins (100 µg) extracted from indicated strains were electrophoretically resolved, transferred to an Immobilon-P membrane, and probed with monoclonal anti-*S. cerevisiae* Gas1p antiserum.

As shown in Figure 8, in *Cacwh8∆*/*Cacwh8∆* mutant the Phrp protein migrates as a diffuse band of 70–130 kDa, representing diverse partially glycosylated forms, as opposed to a single 115 kDa band corresponding to a fully glycosylated protein, as observed in the wild-type CAI4 control. Removal of the *N*-linked glucans by Endo H treatment yielded a protein band of 70 kDa representing the Phr1p polypeptide devoid of *N*-linked glycans. However, in the *Cacwh8∆*/*Cacwh8∆* mutant, some additional forms producing a more diffuse band were still visible. 

**Figure 9:**
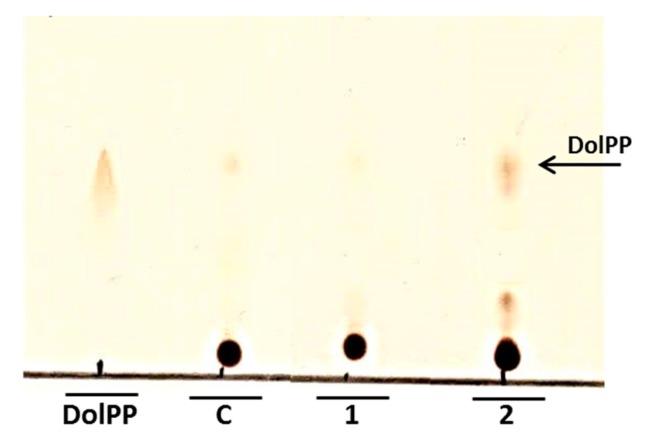
TLC analysis of DolPP in the extracts from the wild type *C. albicans* strain CAI4 (Line C) and *CaCWH8*/*Cacwh8∆* (Line 1) and *Cacwh8∆*/*Cacwh8∆* (Line 2) mutants. Samples (25 µg of dry extracts each) were analyzed on Silica Gel 60 plate as described in Methods. Line DolPP line contains 2 ng of DolPP standard. Arrow indicates the DolPP spot in the extract from *Cacwh8∆*/*Cacwh8∆* mutant.

The Phr1p protein was under *N*-glycosylated despite the higher activities of enzymes located at the beginning of the *N*-glycosylation pathway. This result suggests that blocking of *N*-glycosylation occurs further down the pathway. *N*-glycosylation could be partially inhibited by the increased level of DolPP as was implied by Fernandez et al. [4]. Our analysis confirmed that DolPP is accumulated in the *Cacwh8∆*/*Cacwh8∆* strain (Figure 9). 

### 2.5. Cell Wall Structure in the CaCWH8 Defective Mutants

**Figure 10:**
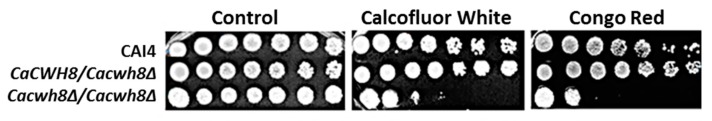
Sensitivity of the wild type *C. albicans* strain CAI4 and the *CaCWH8* mutants to Calcofluor White and Congo Red.

The *S. cerevisiae cwh8∆* mutant shows an increased sensitivity to agents known to interfere with cell wall biogenesis, such as Calcofluor White and Congo Red [21] (Figure 1), indicating that deletion of the *CWH8* gene perturbs cell wall composition. The *C. albicans Cacwh8∆*/*Cacwh8∆* strain, but not the hemizygous one, was also sensitive to Calcofluor White and Congo Red (Figure 10).

Three microliters of a serial 1:10 dilution (starting with 1 × 10^7^ cells) of indicated strains grown in liquid medium were plated on YPG agar plates supplemented with 40 µg/mL uridine (control), and 5 µg/mL Calcofluor White or 5 µg/mL Congo Red and cultivated at 30 °C for 72 h.

**Figure 11:**
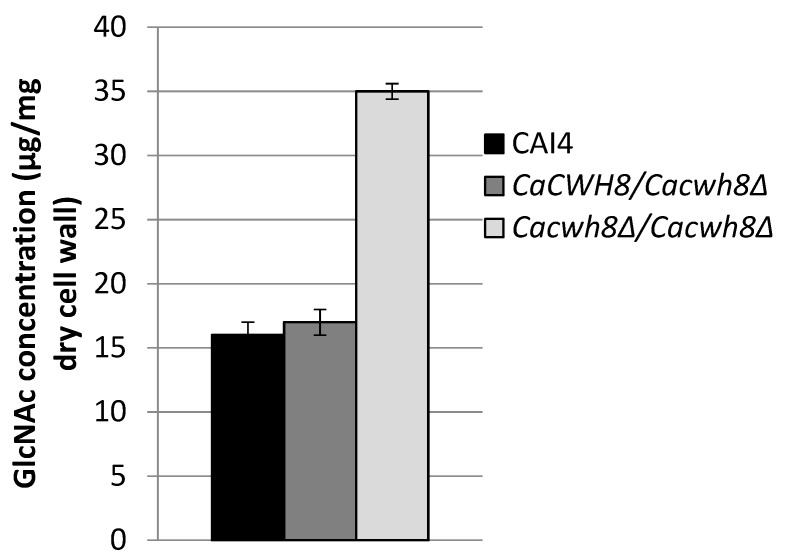
Cell wall chitin content in the control *C. albicans* strain CAI4 and the *CaCWH8* mutants.

To understand the reasons of the increased sensitivity of the *Cacwh8∆*/*Cacwh8∆* mutant toward the cell-wall-perturbing agents, its cell wall composition was determined. In the *Cacwh8∆*/*Cacwh8∆* strain, the chitin content was increased by over 40% compared to the wild type (Figure 11). This likely increased the binding of Calcofluor White interfering with the cell wall assembly [22]. 

Chitin was determined as free GlcNAc after hydrolysis of the cell wall with 6% KOH and enzymatic digestion with chitinase.

Major monosaccharides released from the cell wall by hydrolysis in 2 M TFA, glucose (Glc), mannose (Man), and glucosamine (GlcN) were determined using high performance anion-exchange chromatography. Data are mean ± standard deviation from three independent experiments, each determined in triplicate.

**Figure 12:**
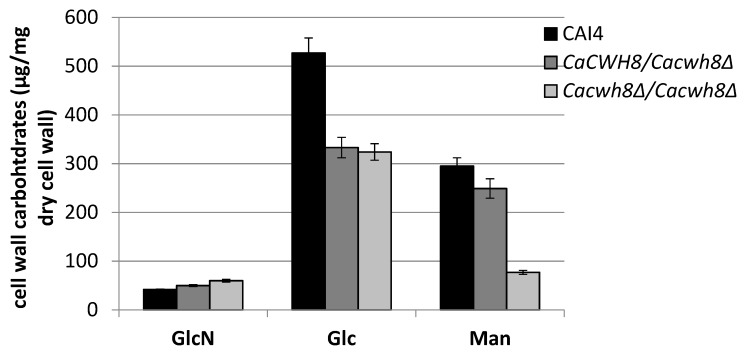
Cell wall carbohydrate composition of wild type CAI4 strain and the *CaCWH8* mutants.

Parallel with an increased content of glucosamine (GlcN), the cell wall of the *Cacwh8∆*/*Cacwh8∆* mutant contained by 40% less glucose (Glc) and 3.8-fold less mannose (Man) compared to the wild type (Figure 12).

### 2.6. Biofilm Formation and Filamentous Growth of the CaCWH8 Mutants

**Figure 13:**
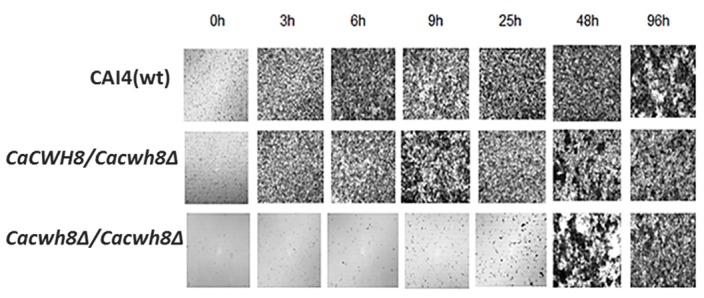
Biofilm formation in the wild type (CAI4) strain and the *CaCWH8* mutants.

The aberrant cell wall composition in the *CaCWH8* deleted strains could influence their ability to form biofilm and/or hyphae. To verify the former possibility, the mutants and the control CAI4 strain were cultivated on Petri dishes (see Materials and Methods), the nonadherent cells were washed away, and the biofilm left was examined under a light microscope. After 3 h of cultivation, biofilm was formed efficiently by the CAI4 cells and the hemizygous mutant, while the double mutant *Cacwh8∆*/*Cacwh8∆* required a much longer period to even begin forming the biofilm (Figure 13).

Three milliliters of cell suspension (1 × 10^6^ cells per mL) was transferred onto sterile 55-mm Petri dishes and incubated at 37 °C. Nonadherent cells were washed away and biofilm was dried and photographed under a light microscope with 10× magnification 

**Figure 14:**
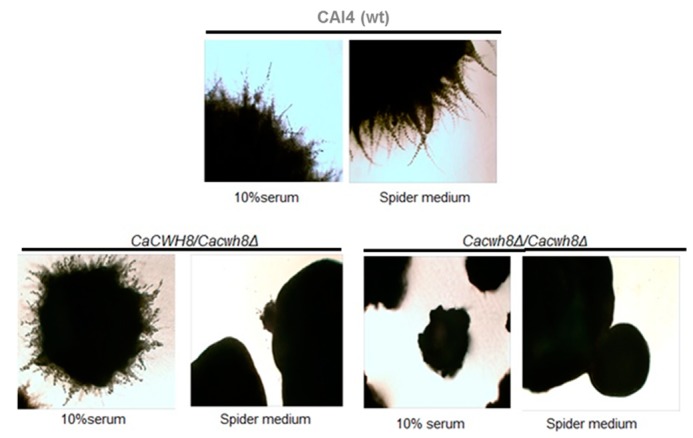
Hyphal growth of *C. albicans* wild-type strain CAI4 and the *CaCWH8* mutants.

To check the ability to form hyphae, the three strains were cultivated in hyphae-inducing conditions, i.e., in the presence of 10% horse serum and on solid Spider medium. The wild type strain formed hyphae on both media, the hemizygote did so on horse serum only, while the double mutant failed to produce hyphae in either conditions (Figure 14).

Hyphae formation was induced for seven days at 30 °C on 10% serum, and on Spider medium and colonies were photographed under a light microscope with 40× magnification.

## 3. Discussion

It is well known that DolP, a glycosyl carrier lipid, is essential for glycosylation of proteins [8,15,23,24,25,26,27]. Notably, the dolichol-dependent glycosylation has been shown to be important for the functioning of *C. albicans* and the fungus–host interactions and consequently, to play a key role in pathogenesis [10,12,28,29,30]. We have previously established that a diminished level of dolichol due to the suppression of the *CaRER2* gene impairs *N*-glycosylation of the Phr1p/Phr2p glycoproteins and perturbs morphogenesis of *C. albicans* [10]. Similar morphological effects were observed upon suppression of one of the genes encoding the DolPPGlcNAc_2_ transferase (Alg13p) involved in *N*-glycosylation [12].

In the present work we found that *N*-glycosylation is also altered upon deletion of the *CaCWH8* gene encoding DolPP phosphatase, bringing about similar effects in hyphae and biofilm formation. However, we also noted quite unexpected consequences of the *CaCWH8* deletion. The compromised recovery of DolP from DolPP in the *Cacwh8∆*/*Cacwh8∆* strain modulated the de novo synthesis of dolichol. The overall *cis*-PTase activity was upregulated and the cellular pool of dolichol elevated. At the gene expression level, the individual *cis*-PTases were affected differently: expression of *CaRER2* and *CaNUS1* dropped several-fold, while the expression of *CaSRT1* increased to a similar extent. 

It has been demonstrated earlier [13,31,32] that the *cis*-PTase encoded by the *SRT1* gene produces polyprenols longer than that of the Rer2p *cis*-PTase. In accordance, longer dolichols (19 and 20 isoprene units) were more abundant in the *Cacwh8∆*/*Cacwh8∆* strain. 

Although the level of dolichols in the *CaCWH8*-deleted mutant was elevated, it was not sufficient to support full glycosylation of the Phrp protein. 

In an *S. cerevisiae rer2∆* strain impaired in glycosylation of carboxypeptidase Y, an overexpression of *SRT1* restored the glycosylation [13]. In that strain, the level of dolichol was markedly increased while the DolP level was low, but high enough to restore the glycosylation of the examined protein [13]. Notably, the *rer2∆* and *cwh8∆* mutants differ in their ability to recover DolP from DolPP. In fact, the utilization of DolPP seems to be crucial for the activity of oligosaccharyltransferase [4]. 

Our *Cacwh8∆*/*Cacwh8∆* strain had a significantly higher level of dolichol compared to the wild type strain and an increased level of DolPP which could not be converted back to DolP due to the lack of CaCwh8p.

In the *S. cerevisiae cwh8∆* mutant, the DolPP level was significantly elevated compared to the wild type [4]. Those authors suggested that the accumulated DolPP hampered the transfer of Glc_3_Man_9_GlcNAc_2_ from DolPPGlcNAc_2_Man_9_Glc_3_ to polypeptide acceptors by inhibiting the activity of the relevant oligosaccharyltransferase [4]. This hypothesis is supported by our results. We observed impaired *N*-glycosylation of a model Phrp protein despite elevated activity of the early steps of *N*-glycosylation. The elevated activities of DPM synthase and GlcNAc transferase in the *Cacwh8∆*/*Cacwh8∆* strain determined without addition of DolP to the reaction mixture indicate that the excess of dolichol synthesized de novo was efficiently phosphorylated and provided ample lipid carrier of mannose, and that the first step of *N*-glycosylation catalyzed by GlcNAc transferase was unaffected. Thus, the inhibition of *N*-glycosylation must occur at a later step of the pathway. In addition, partial inhibition of *N*-glycosylation could have been replaced by *O*-linked glycans. The diffuse Phrp band of 70–130 kDa observed in the *Cacwh8∆*/*Cacwh8∆* strain (Figure 8) suggests that Phrp is extensively *O*-glycosylated and the *O*-linked glycans caused smearing of the Phrp band after the Endo-H teratment. 

The defective *N*-glycosylation in the *Cacwh8∆*/*Cacwh8∆* mutant altered the cell wall composition, most notably by decreasing its content of mannose. It has been demonstrated that mannosylation in *Candida* is a crucial determinant of the cell wall structure and cell surface recognition by the host’s immune system [33]. Perturbation of the cell wall composition often results in compensatory alterations in the cell wall, including activation of chitin synthesis [34]. Such an effect was also observed in our mutant, in which the amount of chitin in the cell wall was significantly elevated compared to the parental strain with a simultaneous decrease of glucans. This caused the *Cacwh8∆*/*Cacwh8∆* mutant to be sensitive to Calcofluor White and Congo Red, agents affecting the cell wall integrity [35]. The inhibitory growth effect of Calcofluor White observed for the mutant is associated with an increased rate of chitin synthesis [36], while Congo Red is known to form a complex with β-1-3-glucan, a main structural component of the fungal cell wall, thereby altering the cell wall assembling [37,38].

Proper protein glycosylation has been demonstrated to be important for the expression of major determinants of pathogenicity, such as the yeast-to-hyphae transition and formation of biofilm in *Candida* [10,12,28,29,30]. A delay in biofilm formation and impaired hyphal growth were observed for the *Cacwh8∆*/*Cacwh8∆* mutant. A similar effect was obtained in the *alg13∆:hisG*/*TR*p*-ALG13* mutant impaired in the initial step of *N*-glycosylation [12] as well as in *Candida* cultivated with tunicamycin, an inhibitor of *N*-glycosylation [39,40]. Since biofilm formation is connected with hyphal growth [41], which was inhibited in our mutant, the difficulties to form biofilm by the mutant were expected.

In summary, in this study we have shown that deletion of *CaCWH8*, encoding the phosphatase converting DolPP to DolP, in *C. albicans* affects expression of genes encoding *cis*-PTases and elevates de novo production of dolichol. Despite the increased activities of DPM synthase and GlcNAc transferase, the initial reactions in the *N*-glycosylation pathway, the *N*-glycosylation of the model protein Phrp was impaired. These observations suggest that the impaired *N*-glycosylation could result from the inhibition of the oligosaccharyltransferase activity by its final product, DolPP.

We have also confirmed a key role of *N*-glycosylation for the proper cell wall composition and for the expression of pathogenic futures such as yeast-to-hyphae transition and biofilm formation in *C. albicans*.

## 4. Materials and Methods

### 4.1. Strains and Growth Conditions

*C. albicans* strain CAI4 (genotype: *ura3∆: imm434/ura3∆: imm434*), a uridine auxotroph (a kind gift from prof. Joachim Ernst, Düsseldorf, Germany), was used for deletion of the *CWH8* (*orf19.3682*) gene. *S. cerevisiae* strain BY4741 (genotype: *MAT**a** his3Δ1 leu2Δ0 met15Δ0 ura3Δ0 cwh8: kanMX4*) was used for expression of *CWH8* gene from *C. albicans*. The *C. albicans* and *S. cerevisiae* strains constructed as well as the plasmids and primers used in the present study are described in Tables S1–S3.

*Escherichia coli* strain DH5αF’ (*F′ supE44 ΔlacU169 {ϕ80 lacZΔM15} hsdR17 recA1 endA1 gyrA96 thi-1 relA1*) was used for plasmid propagation and DNA cloning. *E. coli* was grown at 37 °C in liquid or solid LB medium (1% bacto-peptone, 0.5% yeast extract, 1% NaCl, solidified by 2% agar) supplemented with ampicillin (100 µg/mL) when indicated.

*C. albicans* and *S. cerevisiae* strains were routinely grown on YPG medium (1% yeast extract, 1% bacto-peptone, 2% glucose) or SD medium (0.67% yeast nitrogen base, 2% glucose) supplemented with uridine when required. The *GAL1* promoter was induced on YPGal medium (1% yeast extract, 1% bacto-peptone, 2% galactose).

Spider medium (1% nutrient broth, 1% mannitol, 0.2% K_2_HPO4 and 1.35% agar) and YPSerum (1% yeast extract, 0.5% bacto-peptone, 10% horse serum, 2% agar, if needed) were used for testing hyphae formation [42]. FOA plates (0.67% yeast nitrogen base, 1% casamino acids, 2% glucose, 0.3% 5-fluoroorotic acid, 40 μg/mL uridine, 2% agar) were used to force the excision of the *URA3* gene from *C. albicans* transformants.

### 4.2. Molecular Biology Methods

Chromosomal DNA was isolated from *C. albicans* using the Promega Wizard Genomic DNA Purification kit. Other molecular biology procedures were performed according to standard protocols [43].

### 4.3. S. cerevisiae Cwh8∆/CaCWH8 Strain Construction

In order to complement the *S. cerevisiae cwh8Δ* mutant, the 750 bp coding region of *CaCWH8* was amplified from *C. albicans* genomic DNA using mycCWH8F and mycCWH8R primers, subcloned into pGEM-T Easy and ligated into XhoI/HindIII sites of the pESC yeast vector under the control of the *GAL1* promoter. The obtained plasmid pESC GAL1p-CaCWH8 was used for transformation of *S. cerevisiae cwh8∆* strain.

### 4.4. Transformation of S. cerevisiae Cwh8∆

For yeast transformation, the one-step transformation method of Chen et al. [44] was used. Briefly, stationary yeast culture (24 h) in YPG medium (1.5 mL) was pelleted, resuspended in 80 μL of transformation buffer (200 mM lithium acetate, 40% PEG 4000, 100 mM DTT), mixed with 5 μL of salmon sperm DNA (10 mg/mL) and between 50 ng and 1 µg of transforming DNA, incubated at 45 °C for 30 min, then mixed with 500 μL of water and plated on selective medium.

### 4.5. C. albicans Cacwh8∆/Cacwh8∆ Strain Construction

The double deletion strain *Cacwh8∆*/*Cacwh8∆* was constructed by the URA-blaster method [45]. To construct the deletion cassette for *CaCWH8*, the following pairs of primers were used: CWH8F1F/CWH8F1R to amplify 5′ region of homology (−689 to −297, relative to the first nucleotide in the ORF) and CWH8F2F/CWH8F2R for amplification of the 3′ region of homology (−209 to +121, relative to the last nucleotide in the ORF). Respective fragments were cloned into the KpnI/BglII and BamHI/PstI restriction sites in p5921. The URA-blaster cassette was released by digestion with KpnI and PstI and flanked by the 392 bp upstream and 330 bp downstream sequences complementary to *CaCWH8*. The KpnI/PstI deletion cassette was used for two rounds of gene replacement in the control strain (CAI4). The *URA3* selective marker was purged from the first-round *CaCWH8*/*Cacwh8: hisG-URA3-hisG* mutant by selection on SD supplemented with 0.3% 5-fluoroorotic acid and uridine (40 µg/mL) to obtain a *CaCWH8/Cacwh8: hisG* hemizygous strain. The homozygous deletion strain was then obtained using a deletion cassette constructed with pairs of primers CWH8F3F/CWH8F3R and CWH8F4F/CWH8F4R amplifying 5′ (−210 to +182) and 3′ (+375 to +470) regions of *CaCWH8*). The obtained fragments were cloned into the KpnI/BglII and BamHI/PstI restriction sites in p5921, respectively. The URA-blaster cassette flanked by the 392 bp upstream and 328 bp downstream sequences complementary to *CaCWH8* was cleaved as in the first round and was transformed into the *CaCWH8*/*Cacwh8: hisG* mutant. Integration of the cassette was verified by PCR with primers CWH8F, CWH8R, FverhisG and RverhisG, and by Southern blotting. The *URA3* marker was removed as above.

For Southern blotting and PCR analysis, DNA was isolated from the *CaCWH8*/*Cacwh8: hisG-URA3-hisG*, *CaCWH8*/*Cacwh8∆* and *Cacwh8∆*/*Cacwh8∆* mutants and the CAI4 parental strain (Figure S1). DNA from the two hemizygous mutants was digested with XapI, electrophoresed, transferred to Hybond-N membrane, and hybridized with a DIG-labelled 392 bp probe amplified by PCR with primers CWH8F1F and CWH8F1R on *C. albicans* genomic DNA and visualized with the NBT/BCIP system (Promega, Madison, WI, USA). DNA from the *Cacwh8∆*/*Cacwh8∆* mutant was digested with BclI and hybridized with a 328 bp probe synthesized using primers CWH8F4F and CWH8F4R. CAI4 DNA digested as appropriate served as a negative control.

### 4.6. Transformation of C. albicans 

A standard protocol was used [46]. *C. albicans* culture was incubated overnight at 30 °C until OD_600_ = 0.8–1 and aliquots of 4–6 mL were taken. Cells were collected by centrifugation, washed in LATE buffer (0.1 M lithium acetate, 10 mM Tris-HCl, pH 7.5, 1 mM EDTA pH 7.5) and resuspended in 0.3 mL of LATE. Then, 0.1 mL of cell suspension was mixed with 5 µL of salmon sperm DNA (10 mg/mL) (Sigma-Aldrich, St. Louis, Missouri, USA) and between 1 and 5 µg of transforming DNA and incubated at 30 °C for 30 min. Then, 0.7 mL of PLATE buffer (40% polyethylene glycol 3350 in LATE buffer) was added, the tube was vortexed briefly and incubated at 30 °C overnight. Subsequently, the mixture was heat-shocked at 42 °C for 1 h, cells were collected, washed with sterile water, resuspended in 0.2 mL of water or TE buffer (100 mM Tris-HCl, pH 8.0, 10 mM EDTA, pH 8.0) and plated on selective medium.

### 4.7. Quantitative Reverse Transcription PCR (RT-qPCR)

Total RNA was obtained from *CaCWH8*/*CaCwh8∆* and *Cacwh8∆*/*Cacwh8∆* mutants and the CAI4 parental strain using the single-step method described by Chomczynski and Sacchi [47]. Reverse transcription was performed using iScript Advanced cDNA Synthesis Kit for RT-qPCR (Bio-Rad) and standard protocol. qPCR assays were performed in a Light Cycler 1.6 Instrument (Roche Life Science). For the amplification, Light Cycler Fast Start DNA Master PLUS SYBR Green (Roche Life Science) mix was combined with 0.2 μM forward and reverse primers (Table S2) and cDNA diluted 1:5 with nuclease-free water. The thermal cycling conditions were as follows: initial denaturation at 95 °C for 10 min, followed by 40 cycles of denaturation at 95 °C for 10 s, annealing at 58 °C for 10 s and elongation at 72 °C for 1 s per 25 bp. All primer pairs produced a single amplicon with a uniform melting curve as determined by the denaturation profile of the product.

Two technical repeats were carried out for each data point. The relative expression software tool REST-MCS ©-version 2 [48] was used to quantitate the relative mRNA levels of the selected genes. Data normalization was carried out against the transcript of a reference gene. Among the genes tested, *CPA1* appeared to be the most stable and was used as the reference gene.

### 4.8. Phenotype Analysis

To test yeast strains for sensitivity to various xenobiotics, 3 µL of serial 1:10 dilutions (starting at 1 × 10^5^ cells) of exponentially growing cultures were spotted on YPG agar plates supplemented with uridine (40 μg/mL) and indicated doses of various agents and incubated for 72 h at 30 °C.

### 4.9. Biofilm Formation

*C. albicans* biofilm development was studied under a light microscope [41,49,50]. Cells from overnight culture in YPG medium supplemented with uridine (40 µg/mL), when appropriate, were harvested, suspended in YPG and the optical density at 600 nm was adjusted to a final concentration of 1 × 10^6^ cells per mL. Three-milliliter portions of the standardized cell suspension were transferred into sterile 55 mm Petri dishes and incubated at 37 °C for 3, 6, 9, 25, 48, and 96 h. Subsequently, nonadhered cells were washed away from the biofilm with two 2 mL portions of phosphate buffered saline (PBS) and the biofilm was allowed to dry and examined under a light microscope (Delta Optical). The assay was carried out in triplicate for each strain.

### 4.10. Western Blot Analysis

To detect *C. albicans* Phr proteins (Gas1p homologue) Western blotting was performed essentially as described in Juchimiuk et al. [10]. In brief, cells grown to the late logarithmic phase were harvested by centrifugation and cell extract was obtained by vortexing the cells with glass beads in lysis buffer (25 mM Tris-HCl, pH 7.5, 150 mM NaCl, 1% Nonidet P-40, 0.1% SDS, 1 mM PMSF, 1 mM benzamidine hydrochloride hydrate, 5 mM EDTA, pH 7.5, 1 mM 2-mercaptoethanol, protease inhibitor cocktail (Thermo Fisher Scientific, Waltham, MA, USA). The homogenate was clarified by centrifugation at 10,000× *g* for 10 min. Proteins (100 µg) were separated on 8% SDS-polyacrylamide gel (SDS-PAGE) and transferred to an Immobilon-P (Millipore, Billerica, MA, USA) membrane. The membrane was incubated with rabbit primary anti-Gas1 antibody (1:5000; a kind gift from dr. H. Riezman, Basel) or mouse primary anti-actin antibody (1:5000, Millipore) and then, after washing 6 × 3 min in TBS plus 0.005% Tween 20, with horseradish peroxidase-conjugated anti-rabbit secondary antibody (1:5000, Dako). The results were visualized by chemiluminescent substrate (Super Signal West Pico Chemiluminescent Substrate for HRP-conjugated antibody, Thermo Fisher Scientific, Waltham, MA, USA).

### 4.11. Isolation of Membrane Fraction

*C. albicans* was grown to OD_600_ = 1–1.5, harvested and the pellet was resuspended in two volumes of 50 mM Tris-HCl, pH 7.4 containing 15 mM MgCl_2_ and 9 mM 2-mercaptoethanol. The cells were broken by vigorous vortexing with 0.5 mm glass beads and the homogenate was centrifuged at 10,000× *g* for 10 min at 4 °C to remove unbroken cells and cell debris. The supernatant was then centrifuged at 50,000× *g* for 1.5 h at 4 °C. The pellet was re-suspended in 400 µL of 50 mM Tris-HCl, pH 7.4 containing 350 mM MgCl_2_ and 6 mM 2-mercaptoethanol and homogenized in a tissue grinder. Aliquots were stored at −80 °C.

### 4.12. Enzymatic Activity in C. albicans Cells

#### 4.12.1. Cis-Prenyltransferase Activity Assay

The activity was measured as in [10]. *C. albicans* was grown in yeast nitrogen base selective medium at 28 °C to OD_600_ = 1–1.5. The activity was assayed in the membrane fraction by incubation (final volume 250 μL) of 500 μg of membrane protein with 4 μg FPP, 50 mM sodium phosphate buffer pH 7.4, 0.5 mM MgCl_2_, 20 mM β-mercaptoethanol, 10 mM KF and 3 × 10^5^ cpm [^14^C] IPP (specific activity 52 Ci/mol, American Radiolabeled Chemicals, Inc., St. Louis, MO, USA). After 90 min incubation at 30 °C, the reaction was terminated by addition of 4 mL of chloroform–methanol (3:2 *v*/*v*). The protein pellet was removed by centrifugation and the supernatant was washed three times with 1/5 volume of 10 mM EDTA in 0.9% NaCl. The organic phase was concentrated under a stream of nitrogen and subjected to thin-layer chromatography on HPTLC RP-18 plates (Merck Group, Darmstadt, Germany) developed in 50 mM H_3_PO_4_ in acetone. The zone containing the radiolabeled polyprenols was scraped off and the radioactivity was measured in a scintillation counter [25,51].

#### 4.12.2. Dolichyl Phosphate Mannose (DPM) Synthase Activity Assay

The enzyme activity was assayed in a total volume of 50 µL containing 100 µg of membrane fraction protein, 1 × 10^5^ cpm of GDP[^14^C] mannose (sp. act. 55 Ci/mol, American Radiolabeled Chemicals, Inc., St. Louis, MO, USA) in 40 mM Tris HCl pH7.4 with 10 mM MgCl_2_ and 0.1% Nonidet NP-40 [52]. The reaction was carried out for 5 min at 30 °C and stopped by addition of 4 mL of chloroform–methanol (3:2, *v*/*v*). The mixture was washed once with 1/5 volume of 4 mM MgCl_2_ and twice with 4 mM MgCl_2_ in chloroform: methanol: water (3:48:47, *v*/*v*/*v*). Radioactive dolichyl phosphate mannose formed was measured in a scintillation counter.

#### 4.12.3. *N*-Acetylglucosaminyl (GlcNAc) Transferase Activity Assay

The activity was measured in the membrane fraction by incubation (final volume 50 µL) of 250 µg of membrane protein with 1 × 10^5^ cpm of UDP-*N*-acetyl-D-glucosamine [glucosamine-^14^C(U)] (sp. act. 300 Ci/mol, American Radiolabeled Chemicals, Inc., St. Louis, MO, US) in 40 mM Tris-HCl, pH 7.4, 10 mM MgCl_2_ and 0.1% Nonidet NP-40 at 30 °C for 30 min [53]. The reaction was stopped by addition of 4 mL of chloroform: methanol (3:2, *v*/*v*) and the mixture was washed once with 1/5 volume of 4 mM MgCl_2_ and twice with 4 mM MgCl_2_ in chloroform: methanol: water (3:48:47, *v*/*v*/*v*). Radioactive dolichyl diphosphate *N*-acetylglucosamine and dolichyl diphosphate chitobiose was measured in a scintillation counter.

### 4.13. Dolichol Extraction and HPLC Analysis

For dolichol extraction, *C. albicans* cells from a logarithmic growth phase were disintegrated by vigorous vortexing with 0.5 mm glass beads. The debris was discarded by centrifugation at 10,000× *g* for 10 min and the supernatant was extracted with a chloroform: methanol mixture (chloroform: methanol: cell extract, 1:1:0.3, *v*/*v*/*v*) at 37°C for 90 min. The mixture was brought to the proportion of 3:2:1 (*v*/*v*/*v*) by adding chloroform and disruption buffer and centrifuged for 5 min at 1000× *g* to separate phases. The protein interphase and the aqueous upper layer were removed, the organic phase was washed twice with 10 mM EDTA in 0.9% NaCl concentrated under a stream of nitrogen and hydrolyzed for 90 min in 15% KOH (*w*/*v*) in methanol: water (10:1, *v*/*v*). Subsequently, extraction with diethyl ether was performed twice. The organic solvent was removed under nitrogen, dry lipid residues was dissolved in hexane and applied on a silica column (Silica gel 60, mesh 0.04–0.06 mm). The column was washed with 3% diethyl ether in hexane and dolichols were eluted with 17% diethyl ether in hexane. The solution was dried and dolichols were dissolved in hexane. Samples were applied onto an HPLC Hypersil ODS reversed phase column (4.6 × 60 mm, Hewlett-Packard). For elution a linear gradient of methanol: water (9:1, *v*/*v*) to methanol: isopropanol: hexane (2:1:1, *v*/*v*/*v*) was used and polyprenols were detected at 210 nm. The amount of polyisoprenoids was estimated by comparison with internal Dol_13_ standard added to the cells before the hydrolysis.

### 4.14. DolP and DolPP Determination

For DolP and DolPP extraction and analysis, the method of Rush et al. [6] was used with some modifications. *C. albicans* cells (50 g) were extracted with a chloroform: methanol mixture (chloroform: methanol: cell extract, 1:1:0.3, *v*/*v*/*v*) for two days at RT. Then, chloroform and methanol were added to obtain a final chloroform: methanol: water ratio of 3:2:1 (*v*/*v*/*v*). The upper (aqueous) phase was removed and organic (lower) phase was dried and deacylated in 1 mL of toluene: methanol (1:1, *v*/*v*) containing 0.1 M KOH on ice for 30 min. After neutralization with acetic acid, 5 mL of chloroform: methanol mixture (2:1, *v*/*v*) was added. Organic (lower) phase was washed with 1 mL of water and then with 1 mL of chloroform: methanol: water (3:48:47, *v*/*v*/*v*) and dried. The resultant residue was dissolved in chloroform: methanol (2:1, *v*/*v*). Crude lipid mixture was loaded onto a DEAE-Sephadex (acetate form) ion exchanger column and DolP and DolPP were separated from neutral lipids by elution with chloroform: methanol (2:1, *v*/*v*). Fractions were analyzed by TLC on Silica gel 60 plates developed with chloroform: methanol: water (65:25:4, *v*/*v*/*v*) and visualized with iodine vapor. Plate was scanned using Epson Perfection V700 Photo scanner.

### 4.15. Isolation of Cell Wall Fraction

*C. albicans* was cultivated in YPD medium, washed with 10 mM Tris-HCl, pH 7.5, suspended in the same buffer, disintegrated by vigorous vortexing with 0.5 mm glass beads in the presence of a protease inhibitor cocktail (Sigma-Aldrich) and centrifuged at 1500× *g* for 10 min. The resulting pellet containing cell walls was washed with ice-cold 1 M NaCl until disappearance of absorbance at 260–280 nm [54]. The preparation was then washed with miliQ water to remove salt and lyophilized.

### 4.16. Isolation and Analysis of Cell Wall Components

For chitin determination, the cell wall preparation was incubated in 6% KOH for 90 min at 80 °C in order to release cell wall proteins. After neutralization with acetic acid, the pellet was washed with PBS and a buffer containing 18 mM citric acid and 60 mM dibasic sodium phosphate, pH 6.0. Then the pellet was treated with chitinase C (InterSpex Products, ChemNet, Zhejiang, China) for 3 h at 37 °C. The *N*-acetylglucosamine liberated was quantitated with Erlich′s reagent as described [55]. For determination of alkali-insoluble β-glucan, the cell wall preparation was treated with 3% NaOH three times for 1 h at 75 °C. After each round of hydrolysis, the sample was centrifuged at 16,000× *g* for 15 min at RT to remove released mannoproteins and alkali-soluble glucan and neutralized by washing twice with 0.1 M Tris-HCl, pH 7.4, followed by 10 mM Tris-HCl, pH 7.4. The alkali-insoluble fraction was digested overnight with 5 mg/mL zymolyase 20T (MP Biomedicals, Inc.) in 10 mM Tris-HCl, pH 7.4 at 37 °C. The material was then centrifuged at 16,000× *g* or 15 min and the supernatant was dialyzed against water. The undigested β-1,6-glucan remaining in the dialysis bag was collected and quantified. Total alkali-insoluble glucan was determined as reducing sugars prior to the dialysis by the method cited above. The alkali-insoluble β-1,3-glucan content was calculated by subtraction of the β-1,6-glucan content from that of total glucan.

To determine the monosaccharide composition of the cell wall constituents, the cell wall preparation was hydrolyzed o/n in 4 M trifluoroacetic acid (TFA) at 100 °C. After cooling on ice, the sample was centrifuged at 17,000× *g* for 5 min at 4 °C and the supernatant was dried under nitrogen and washed twice with pure methanol. After removing methanol with nitrogen, the pellet was resuspended in miliQ water and filtrated through a Millipore Filter Device (0.45 µm pores) by centrifugation at 16,000× *g* for 4 min. Samples were stored at −20 °C. Monosaccharides were determined by high performance anion-exchange chromatography using a Dionex ICS-3000 Ion Chromatography System with a Carbo Pac PA10 analytical column. Neutral sugars were eluted with 18 mM NaOH at 0.25 mL/min [56].

## Supplementary Materials

Supplementary materials can be found at https://www.mdpi.com/1422-0067/20/20/5067/s1.
