# Supplementary material for: Inhibition of Dephosphorylation of Dolichyl Diphosphate Alters the Synthesis of Dolichol and Hinders Protein N-Glycosylation and Morphological Transitions in Candida albicans
Source: Int J Mol Sci. 2019 Oct 12;20(20):5067. doi: 10.3390/ijms20205067 (PMC6829516; doi:10.3390/ijms20205067)
Supplement: Supplementary file 1 [file ijms-20-05067-s001.pdf]

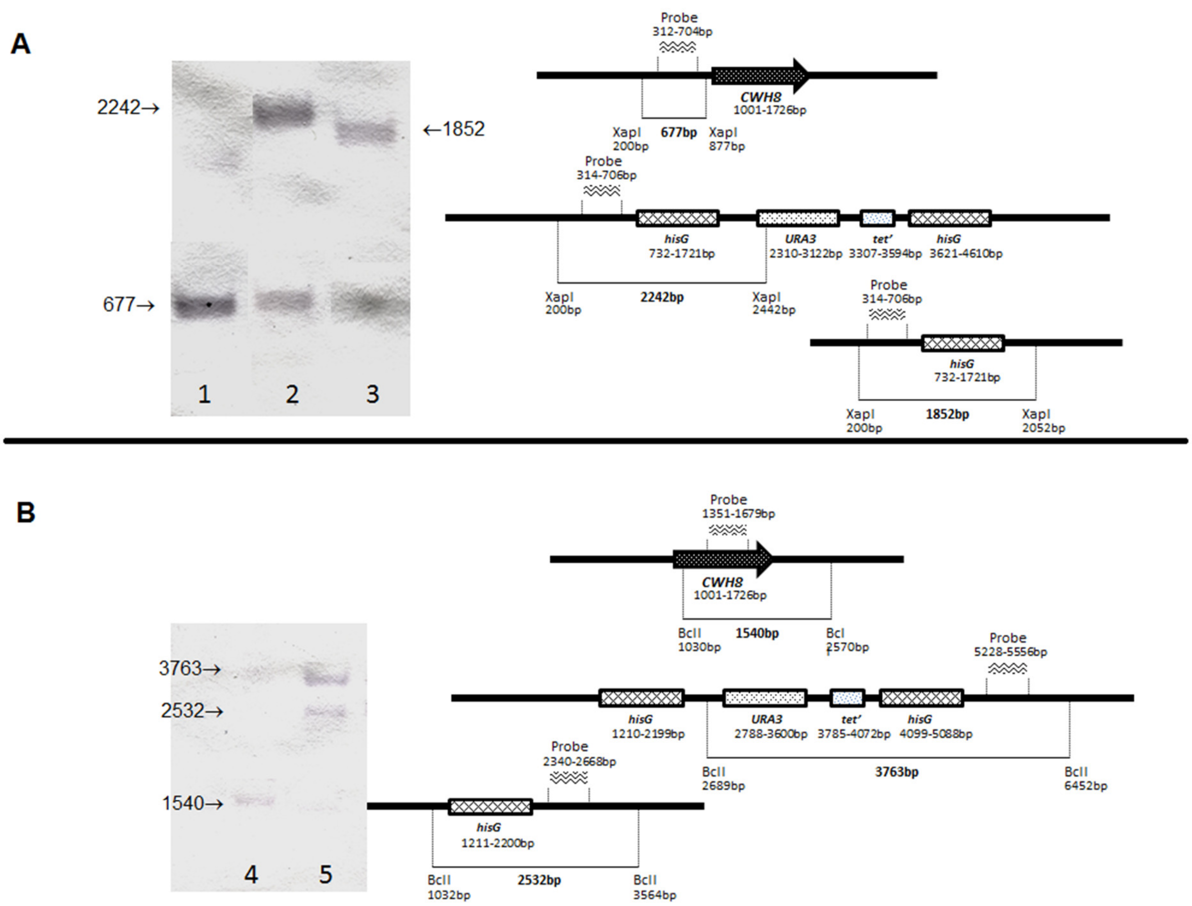

Figure S1 *CaCWH8* organization in *C. albicans* genome

A. DNA from control CAI4 strain (lane 1), and hemizygous mutants *CaCWH8/Cacwh8::hisG-URA3-hisG* (lane 2) and *CaCWH8/Cacwh8Δ* (lane 3) was digested with Xap I and hybridized with 392-bp digoxigenin-labeled *CaCWH8* fragment.
B. DNA from control CAI4 strain (lane 4) and *Cacwh8Δ/Cacwh8Δ* mutant (lane 5) digested with Bcl I and hybridized with the 328-bp digoxigenin-labeled *CaCWH8* fragment.

Table S1 *Candida albicans* and *S. cerevisiae* strains used in this study

Name	Genotype	Source
***C. albicans* CAI4**	*ura3 Δ::imm434/ ura3 Δ::imm434*	Fonzi and Irwin, 1993
C. albicans CaCWH8/Cacwh8Δ	CAI4; *CaCWH8/Cacwh8Δ::hisG*	This study
C. albicans Cacwh8Δ/Cacwh8Δ	CAI4; *Cacwh8Δ:: hisG /Cacwh8Δ::URA3*	This study
***S. cerevisiae* BY4741**	*MATa his3Δ1 leu2Δ0 met15Δ0 ura3Δ0*	Euroscarf
S. cerevisiae* BY4741:*cwh8::kanMX4	*MATa his3Δ1 leu2Δ0 met15Δ0 ura3Δ0cwh8::kanMX4*	Euroscarf
***S. cerevisiae BY4741:cwh8::kanMX4/pESCCaCWH8* URA3**	*MATa his3Δ1 leu2Δ0 met15Δ0 ura3Δ0/URA3 cwh8::kanMX4/CaCWH8*	This study

Table S2 Primers used in this study

Name	Sequence	Remarks
**CWH8 F1F**	TCAGGTACCATTCACAATCTAATCTCC	Restriction site for KpnI underlined
**CWH8 F1R**	ACAAGATCTGGAAGGAAGGAAGTAAAGAA	Restriction site for BglII underlined
**CWH8 F2F**	TAGGATCCATTATAACTTCAGTGGCTAG	Restriction site for BamHI underlined
**CWH8 F2R**	GATCTGCAGAACAAACATGAAAAAGGGTA	Restriction site for PstI underlined
**CWH8 F3F**	TTAGGTACCGTAAATCCAACTCAGCATAT	Restriction site for KpnI underlined
**CWH8 F3R**	CCGAGATCTTAAATGACCACCAACTATAA	Restriction site for BglII underlined
**CWH8 F4F**	TAGGATCCATTATTAAAAGTCCCCATGC	Restriction site for BamHI underlined
**CWH8 F4R**	TTACTGCAGATGCTTCGTTTCTTTCTCTT	Restriction site for PstI underlined
**CWH8F**	TCCCTTGCTTGACTTACTTACT	
**CWH8R**	CAACAAGTCCAACATCTCTAGC	
**FverhisG**	CGATACAGACCGGTTCAGAC	
**RverhisG**	GCGCTTTCAGTTTCTCCATG	
**mycCWH8F**	CTCGAGATGATGTTAGATTATAATCCAGTTCC	Restriction site for XhoI underlined
**mycCWH8R**	AAGCTTCTATTCTCCAAGTGATATATC	Restriction site for HindIII underlined
**Nus1F4**	CACAAGTGATTAGGGAGGACATT	qPCR *NUS1*
**Nus1R4**	GACCATGCTGTCAACTCACTAA	
**Rer2F5**	GTTGTGTGAGGAGTATGGAGTT	qPCR *RER2*
**Rer2R5**	ACACGGCACGAGTATTGTT	
**Srt1F4**	CCCTATACTGCTCGTGATGAAAT	qPCR *SRT1*
**Srt1R4**	ACGATCATGAAGTTCTCCTGAAA	
**Cpa1F2**	GGGAGAATTACGATTGGTGAAGA	qPCR *CPA1* control
**Cpa1R2**	GGTGCCTTGGTGGTTACTTTA	
**Act1F3**	TTGGATTCTGGTGATGGTGTTA	qPCR *ACT1* control
**Act1R3**	TCAAGTCTCTACCAGCCAAATC	

Table S3 Plasmids used in this study

Name	Description	Source
**pGEM-T Easy**	T/A subcloning	Promega
**p5921**	*URA*-blaster deletion	Fonzi and Irwin,1993
**pESC yeast epitope tagging vector**	*GAL1*promoter, *URA3*	Agilent Technologies
